# Editorial: Understanding the mesenchymal to epithelial transition: a much needed angle for epithelial mesenchymal plasticity

**DOI:** 10.3389/fcell.2024.1497515

**Published:** 2024-10-07

**Authors:** Hani Alotaibi, Ralph Meuwissen, A. Emre Sayan

**Affiliations:** ^1^ Izmir International Biomedicine and Genome Institute, Dokuz Eylül University, Izmir, Türkiye; ^2^ Basic and Translational Research Program, Izmir Biomedicine and Genome Center, Izmir, Türkiye; ^3^ Basic Oncology Department, Ege University, Izmir, Türkiye; ^4^ Cancer Sciences Unit, University of Southampton, Southampton, United Kingdom

**Keywords:** epithelial-mesenchymal plasticity (EMP), cancer biology, metastasis, mesenchymal-to-epithelial transition (MET), epithelial-to-mesenchymal transition (EMT)

The epithelial-mesenchymal transition (EMT) and the mesenchymal-epithelial transition (MET) are two critical biological processes with functions that extend beyond their role during development to wound healing and cancer metastasis. However, despite the wealth of research on EMT, MET remains underexplored. Recent studies have highlighted the importance of transcriptional and epigenetic regulation of MET, particularly in cancer biology. This review summarizes key developments in the regulation of MET at the transcriptional level, focusing on findings from recent research, including the Research Topic “*Understanding the Mesenchymal to Epithelial Transition: A Much Needed Angle for Epithelial-Mesenchymal Plasticity*” published in Frontiers in Cell and Developmental Biology.

In this Research Topic, we aimed to enhance our understanding of epithelial-mesenchymal plasticity (EMP), primarily through the lens of the mesenchymal to epithelial transition (MET) and its implications in various biological contexts, especially cancer progression. These studies collectively highlight the complex interplay of molecular pathways and signaling networks that govern EMP, underlining the importance of these transitions in tumor biology and tissue homeostasis. While no particular article in this Research Topic specifically focused on MET, they presented novel insights on EMT from different perspectives.

For example, one article explores how circFGGY, a circular RNA, influences hepatocellular carcinoma by regulating the miR-545-3p/Smad7 axis, ultimately inhibiting cell growth, invasion, and the EMT process (Feng et al.) by functional disrupting the TGFβ pathway. Upregulation of Smad7 by circFGGY caused an increased TGFβ activity, which was then in turn inhibited by miR-545-3p acting as antagonist of both circFGGY and Smad7. This aligns with the existing literature that highlights the role of non-coding RNAs in regulating EMT. Specifically, the microRNA miR-200 family is known to help maintain epithelial characteristics and prevent EMT by targeting key transcription factors like ZEB1 and ZEB2 (Brabletz et al., 2018). Identifying circFGGY as a tumor suppressor suggests that non-coding RNAs could be valuable targets for developing new therapeutic strategies against EMT in cancer.

Another study focuses on identifying EMT-related genes and their prognostic significance in lower-grade gliomas, revealing how specific gene expressions correlate with tumor behavior and treatment responses (Wu et al.). Notably, expression patterns of EMT-related gene panels (EMTsig) revealed potential functional interactions of adjacent non-tumor tissue during EMT and malignant LGG progression. Moreover, EMTsig can have its use as prognostic tool for predicting efficacy of chemo-, targeted drug, and ICB therapies. This finding is consistent with previous research indicating that high levels of EMT-associated genes are often linked to poor prognosis and increased metastatic potential in various cancers (Kalluri and Weinberg, 2009). Recognizing these molecular signatures can aid in tailoring therapeutic strategies to individual patient profiles.

The research on ameloblastin and its interactions with the extracellular matrix (ECM) extends the understanding of EMP beyond traditional concepts (Reseland et al.). By examining how EMT and ECM interplay, this study suggests that manipulating these interactions could yield insights into both tissue regeneration and cancer biology. Previous studies have shown that the ECM plays a critical role in regulating EMT by providing mechanical and biochemical cues that influence cellular behavior (Tzanakakis et al., 2018).

Furthermore, another investigation looks at how EMP influences cancer spreading patterns across different types of tumors (Wang and Yan). This study explains how strong hysteresis influences the MET process to become a slow process, leading to multiple intermediate stages between the complete epithelial and mesenchymal states, thereby indicating that a transient hybrid epithelial/mesenchymal phenotype occurs in some if not most cancer cell types. They propose that EMT is controlled by hysteretic control mechanisms and can be defined in four subtypes with various hysteretic control. This research aligns with established knowledge that the dynamic transition between epithelial and mesenchymal states facilitates cancer cell dissemination and metastasis (Yang and Weinberg, 2008; Celia-Terrassa et al., 2018). Understanding these patterns is vital for developing targeted interventions that consider the plasticity of cancer cells.

Additionally, a review discussing plasticity in cell migration modes across development, physiology, and disease emphasizes how EMP affects cellular behaviors in various contexts (Pourjafar and Tiwari). By exploring different migration modes influenced by EMP, this study highlights the adaptability of tumor cells to environmental cues, which is essential for formulating effective therapeutic strategies against cancer metastasis. Research indicates that cancer cells can switch between different migration modes, such as collective and individual migration, a key aspect of their plasticity that contributes to metastatic potential (Friedl and Gilmour, 2009; Friedl and Alexander, 2011).

Finally, a case report detailing the immunologic and molecular landscape of a unique cancer presentation underscores the complexity of EMP in tumor biology and the need for personalized treatment approaches (Riedinger et al.). This aligns with the growing understanding that the tumor microenvironment, including immune cell interactions, plays a critical role in regulating EMT and cancer progression (Quail and Joyce, 2013). Understanding these interactions will be crucial for developing therapies that effectively target the tumor microenvironment.

While research on epithelial to mesenchymal transition (EMT) is extensive, the mesenchymal to epithelial transition (MET) remains less explored, leaving significant gaps in our understanding. Recent studies have highlighted the roles of key transcription factors like Ovol2, Grhl2, Grhl3, and C/EBPα in promoting MET, which is crucial for re-establishing epithelial characteristics. For example, Ovol2 drives MET by repressing EMT-inducing factors such as Zeb1, facilitating epithelial differentiation (Watanabe et al., 2019). Grhl2 similarly promotes MET by antagonizing EMT and enhancing E-cadherin expression (Werth et al., 2010), while Grhl3 contributes by stabilizing epithelial junctions and promoting epithelial-specific proteins (Alotaibi et al., 2015). C/EBPα also plays a vital role by inhibiting EMT, making it a potential target for cancer therapies aimed at limiting metastasis (Lourenco et al., 2020).

Recent advances in understanding the transcriptional and epigenetic regulation of MET have shed light on the complex molecular networks controlling this critical biological process. Key transcription factors such as Klf4, Grhl2/3, Ovol2, and C/EBPα, as well as non-coding RNAs and epigenetic modifications, play crucial roles in regulating MET and maintaining epithelial characteristics. These findings have significant clinical implications, particularly in the context of cancer therapy and tissue regeneration.

Despite EMT-inducing transcription factor expression in primary tumours remain a good predictor for disease free survival and therapy response, it is still not clear if their expression in maintained in secondary tumours. Classical understanding of metastasis cascade dictates mesenchymal carcinoma cells are the main culprits of recurrence which need to undergo MET to establish tumours at the secondary site (Brabletz et al., 2018). To achieve this objective they need to actively regulate EMT and MET programs. A good example of this phenomenon is Zeb1 expression in primary metastatic colorectal cancer, which is repressed by miR200 family of micro-RNAs at the secondary site (Hur et al., 2013; Burk et al., 2008). On the other hand, the other ZEB family member gene, Zeb2 is expressed in both primary and secondary sites leading to therapy resistance (Sreekumar et al., 2021). Retained expression of Zeb2 probably causes a partial EMT or poise cells to a plastic state. In this case it is unclear how Zeb2 escapes from miR200 dependent gene expression repression or how Zeb2 expressing cancer cells can re-gain E-Cadherin expression. There is clearly a hierarchy and difference in transcription repression capability between EMT inducing transcription factors which should be analysed in the context of EMP.

As research continues to explore the intricate balance between EMT and MET, the potential for therapeutic interventions targeting these transitions becomes increasingly clear. Further research is still needed to highlight the importance of understanding epithelial-mesenchymal plasticity in both normal and pathological contexts.

In summary, this Research Topic advances our understanding of EMP by identifying key regulatory elements, clarifying clinical implications, and highlighting the significance of plasticity in cellular behavior. Ongoing research into these transitions, particularly MET, is essential for deepening our comprehension of tumor biology and may lead to innovative therapeutic strategies to combat cancer.
